# Induced resistance for plant defense

**DOI:** 10.3389/fpls.2015.00109

**Published:** 2015-02-24

**Authors:** Andrés A. Borges, Luisa M. Sandalio

**Affiliations:** ^1^Grupo de Activadores Químicos de Defensa de la Planta, Departamento de Agrobiología, Instituto de Productos Naturales y Agrobiología, Consejo Superior de Investigaciones Científicas, San Cristóbal de La LagunaTenerife, Spain; ^2^Departamento de Bioquímica, Biología Celular y Molecular de Plantas, Estación Experimental del Zaidín - Consejo Superior de Investigaciones CientíficasGranada, Spain

**Keywords:** induced resistance, priming, biotic stress, abiotic stress, priming agents, crop protection, sustainable agriculture

In this century the human being must face the challenges of producing enough to feed a growing population in a sustainable and environmentally friendly way. The yields are with increasing frequency affected by abiotic stresses such as salinity, drought, and high temperature or by new diseases and plagues. The Research Topic on *Induced Resistance for Plant Defense* focuses on the understanding the mechanisms underlying plant resistance or tolerance since these will help us to develop fruitful new agricultural strategies for a sustainable crop protection. This Topic and their potential applications provide a new sustainable approach to crop protection. This technology currently can offer promising molecules capable to provide new long lasting treatments for crop protection against biotic or abiotic stresses.

One of the most studied plant defense inducers and priming agents, the β-aminobutyric acid or BABA, has been used for investigating the transgenerational epigenetic basis of priming defense and the mechanistic of long-lasting induced resistance (Luna et al., 2014a). Interestingly, these authors found that BABA-IR can be detected up to 28 days after treatment of wild-type Arabidopsis through NPR1-dependent resistance but this effect disappear by 14 days after treatment when a NPR1-independent resistance is activated. Another work about BABA (Schwarzenbacher et al., 2014) included in this ebook is a commentary about a previously published paper (Luna et al., 2014b) which study the plant perception of BABA mediated by an aspartyl-tRNA synthetase. Using BABA as priming agent in a screening for Arabidopsis mutants against the biotrophic oomycete *Hyaloperonospora arabidopsidis*, authors identify an impaired in BABA-induced Immunity 1 (*IBI1*) gene, encoding an aspartyl-tRNA synthetase (AspRS). This mutation seems to block both priming SA-dependent or SA-independent responses to BABA, indicating unilateral control of BABA-induced resistance by IBI1 (Luna et al., 2014b).

Nitrogen fertilization influences plant-pathogen interactions and elevated levels of nitrogen can promote susceptibility against biotrophs as well as can influence in plant resistance. The disruption of an ammonium transporter involved in the plant immune system, the ammonium transporter AMT1.1, alters basal defenses generating resistance against *Pseudomonas syringae* and *Plectosphaerella cucumerina* (Pastor et al., 2014a). In this work their authors study the role of this ammonium transporter on the basal defenses and the resistance against *P. syringae* and *P. cucumerina* demonstrating that it is a negative regulator of Arabidopsis defense responses (Pastor et al., 2014a).

Cross-talk between different signaling pathways has been reported to generate both synergistic and antagonistic defense responses. In some cases this cross-talk might contribute to fine-tune defense responses against some pathogens according to its mode of infection. Using some resistance elicitors such as acibenzolar-S-methyl (ASM), β-aminobutyricacid (BABA), cis-jasmone (CJ), and a combination of the three compounds, which involve SA and/or JA-dependent signaling pathways, Walters et al. (2014) study if these treatments are capable to control infection of spring barley by *Rhynchosporium commune* under field conditions (Walters et al., 2014).

Heavy metals like cadmium are an important source of contamination and a serious problem in the modern agriculture. We need further research on this abiotic stress. Some important novel insights into cadmium sensing have been studied and it seems to induce a rapid mobilization of defense mechanisms through the activation of specific signaling transduction pathways (Chmielowska-Bak et al., 2014).

Priming phenomena have been widely described, however mechanisms underlying are still unclear. One of the papers included in this ebook study how during the priming phase plant prepares for further challenges by accumulating and storing conjugates or precursors of molecules as well as other compounds that play a role in defense (Pastor et al., 2014b).

An important issue in this topic is the influence of the environment and the genotype in the plant responsiveness to defense elicitors. Interestingly, Bruce (2014) have shown that herbivore and pathogen attack can promote defense induction phenotypes across generations and that epigenetic change may be the basis for its long lasting effects (Bruce, 2014).

Plants are able to respond to biotic or abiotic stresses through a complex network involving phytohormones, a potent secondary metabolism and secondary messengers like calcium, and stress receptors. Light also plays a key role in plant resistance. Protein kinase/phosphatase cascades are another important component of this network. Rasool and co-workers study the effects of the light on these proteins using light-grown *Arabidopsis thaliana* wild type and in mutant lines defective in several protein phosphatase regulatory subunits on aphid fecundity and susceptibility to *P. syringae* infection (Rasool et al., 2014).

Aranega and co-workers update the role of natural compounds as priming agents and focus on the molecule hexanoic acid as a model. They review on the different mode of action of natural compounds that induce resistance by a priming mechanism (Aranega et al., 2014).

Another interesting work included in this topic focuses on the activation of the plant immunity by a pathogen detection system known as pattern-triggered immunity (PTI) response (Huang and Zimmerli, 2014). This system relies on the accurate detection of pathogen-or microbe-associated molecular patterns by pattern-recognition receptors (PRRs). Resistance is the rule in the majority of plants. Huang and Zimmerli suggest that the reinforcement of PTI through genetic engineering may generate crops with broad-spectrum field resistance (Huang and Zimmerli, 2014).

Finally, Borges and co-workers propose priming crops as a way for controlling biotic and abiotic stresses and focus on the effect of the water-soluble vitamin K3 derivative, known as menadione sodium bisulphite (MSB), as a novel priming agent and as a tool for studying priming mechanisms. The work review the priming phenomenon and the importance of reactive oxygen species (ROS) as key signaling molecules that contribute to control of plant development as well as to the sensing of the external environment and priming induction (Borges et al., 2014).

## Conflict of interest statement

The authors declare that the research was conducted in the absence of any commercial or financial relationships that could be construed as a potential conflict of interest.
